# Nonlinear analysis of heart rate variability signals in meditative state: a review and perspective

**DOI:** 10.1186/s12938-023-01100-3

**Published:** 2023-04-13

**Authors:** Bhabesh Deka, Dipen Deka

**Affiliations:** 1 Department of ECE, School of Engineering, Tezpur University, Assam, India; 2 Department of Instrumentation Engineering, Central Institute of Technology, Kokrajhar, India

**Keywords:** Heart rate variability, Meditation, Phase-space representation, Entropy, Long-range correlation, Dynamical complexity

## Abstract

**Introduction:**

In recent times, an upsurge in the investigation related to the effects of meditation in reconditioning various cardiovascular and psychological disorders is seen. In majority of these studies, heart rate variability (HRV) signal is used, probably for its ease of acquisition and low cost. Although understanding the dynamical complexity of HRV is not an easy task, the advances in nonlinear analysis has significantly helped in analyzing the impact of meditation of heart regulations. In this review, we intend to present the various nonlinear approaches, scientific findings and their limitations to develop deeper insights to carry out further research on this topic.

**Results:**

Literature have shown that research focus on nonlinear domain is mainly concentrated on assessing predictability, fractality, and entropy-based dynamical complexity of HRV signal. Although there were some conflicting results, most of the studies observed a reduced dynamical complexity, reduced fractal dimension, and decimated long-range correlation behavior during meditation. However, techniques, such as multiscale entropy (MSE) and multifractal analysis (MFA) of HRV can be more effective in analyzing non-stationary HRV signal, which were hardly used in the existing research works on meditation.

**Conclusions:**

After going through the literature, it is realized that there is a requirement of a more rigorous research to get consistent and new findings about the changes in HRV dynamics due to the practice of meditation. The lack of adequate standard open access database is a concern in drawing statistically reliable results. Albeit, data augmentation technique is an alternative option to deal with this problem, data from adequate number of subjects can be more effective. Multiscale entropy analysis is scantily employed in studying the effect of meditation, which probably need more attention along with multifractal analysis.

**Methods:**

Scientific databases, namely PubMed, Google Scholar, Web of Science, Scopus were searched to obtain the literature on “HRV analysis during meditation by nonlinear methods”. Following an exclusion criteria, 26 articles were selected to carry out this scientific analysis.

## Introduction

**Fig. 1:**
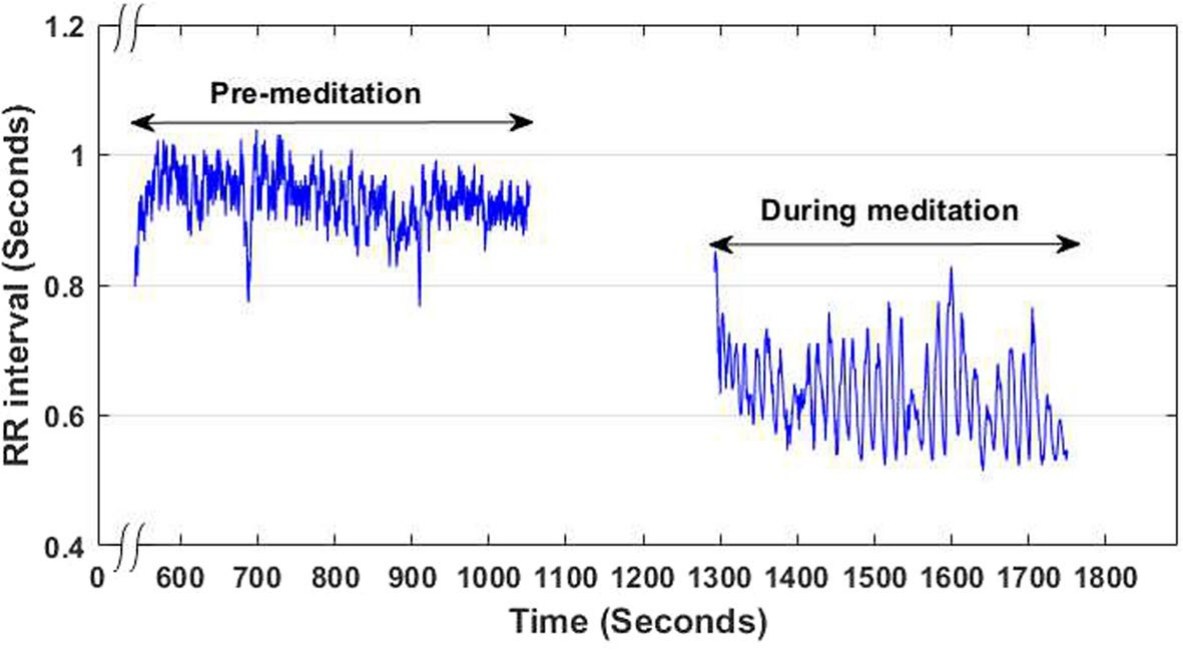
HRV signals of a representative Kundalini Yoga meditator (Y1) before meditation (left) and during meditation (right) [14]

In the last decade, a growing investigation on the role of yoga and meditation, especially on psychological and cardiac health using heart rate variability (HRV) features, has been observed. Relevant research works claim that yoga/meditation not only improves mental calmness and boosts concentration level, but also keeps the cardiovascular system healthy [1–4]. It is also to be noted that HRV signal has received tremendous response from the biomedical research community probably due to its ease of acquisition, noninvasiveness, and direct association with the functioning of the autonomic nervous system (ANS) [5–10]. The ANS plays a very important role in controlling various physiological processes, like the cardiovascular regulation, respiration, thermoregulation, urinary system, digestion, etc., and thus maintaining the homeostasis. Over the last few decades, different works [10–13] have demonstrated that meditation has interesting impact on the activity of the ANS, which functions via its two divisions, namely, the parasympathetic nervous system (PNS) and the sympathetic nervous system (SNS). Under different actions and health conditions, the predominance of these two sub-systems automatically changes. Activation of SNS and PNS causes the release of chemical messengers; epinephrine and norepinephrine in case of the former, while acetylcholine in the latter [5, 10]. These hormones control the firing rate of the sinoatrial (SA) node, responsible for maintaining the HR. Parasympathetic activation decreases HR and sympathetic activation increases HR. This leads to a variation in interbeat (RR) intervals, which is represented by the HRV signal. Changes in HR dynamics under pathological, normal sinus rhythm, and different meditative states are shown in Fig. 1.

Literature study shows that linear (time or frequency domain) HRV analysis methods are more commonly used to distinguish the meditative state and the pre-meditative state. Out of them, time domain methods essentially perform statistical and geometrical calculations using the RR intervals time series. Although they are popularly used for studying the effect of meditation [15–17], they have the limitation of providing only the average information about the signal, while frequency domain methods are used for evaluating the spectral power distribution of the HRV signal in three distinct bands. These bands are classified as very low frequency (VLF), low frequency (LF), and high frequency (HF) bands. It is to be noted that another band, named the ultra low frequency (ULF) band (0$-$0.0033 Hz) is seldom used due to lack of reliable findings of its correlation with the ANS. The significance of VLF (0.0033$-$0.04 Hz) power is its association with thermoregulation, renin–angiotensin system, and peripheral vasomotor activity [18, 19]. LF power (0.04$-$0.15 Hz) reflects the stimulation of both the SNS and PNS with a greater influence from the former. On the other hand, HF power (0.15$-$0.4 Hz) reflects the stimulation of PNS. Based on these measures, an important parameter: the ratio of LF power to HF power is computed, which reflects the sympathovagal balance between the SNS and PNS. The values of these parameters are used to analyze whether there is any impact of meditation of ANS [11, 20–22]. In majority of these frequency domain studies, the fast Fourier transform (FFT) is used to estimate the power spectral density (PSD) for studying the effect of meditation. However, considering the non-stationary behavior of HRV, few [21, 23] of them have resorted to wavelet-based HRV analysis during meditation.

Even though linear methods of HRV analysis are being used in the detection of mind–body interactions, they are not able to perceive the underlying complex patterns in the HRV signal. Meditation causes a change in HRV dynamics arising from respiratory sinus arrhythmia (RSA) as well as the changes in autonomic activity because of concentrated mind. For the study of various phenomena, like self similarity, deterministic chaos, irregularity in patterns, nonlinear tools are assumed to be more effective. Different nonlinear techniques and tools are already popular in various fields including hydrodynamics, mechanical, and electrical engineering, etc. Subsequently, in the study of human physiological conditions also, they are found to be quite effective. The complexity and the dynamical behavior of HRV signals during meditation are mainly analyzed with the help of different nonlinear parameters, including the largest Lyapunov exponent (LLE), the correlation dimension (CD), and the nonlinearity score (NLS) [12, 24, 25]. Besides, several entropy measures, like the Shannon entropy (ShEn), the approximate entropy, the sample entropy, the correlation entropy, the symbolic entropy, the Renyi entropy, the permutation entropy, etc., are also used [13, 26, 27]. The presence/absence of long-range dependence has also been studied in some studies to examine whether any significant changes occur during meditation based on fractal/multi-fractal analysis [15, 28, 29] and visibility graph [28, 30, 31] techniques.

A thorough literature search on “HRV analysis for studying the effects of meditation” returns papers which are primarily focussing on linear analysis techniques and a massive vacuum is observed in the reviews related to nonlinear HRV analysis for studying the impacts of meditation. As numerous original works have demonstrated the nonlinear property of HRV signal, it is of paramount importance to have an analysis on the published papers related to the aforementioned subject. This led us to cumulate the findings of various nonlinear methods of HRV signal to analyze the impacts of meditation, followed by their critical analysis.

In order to provide a critical analysis on the works, we first mention different nonlinear parameters for characterization of HRV dynamics during and before the practice of meditation and yoga. Relevant theoretical backgrounds are briefed to extract specific knowledge and understanding of their usefulness in studying the dynamics of HRV signal. Next, we add our perspectives to aid in the quest for continuous and breakthrough findings on this multidisciplinary research domain.

The rest of the paper is organized as follows. "Results" section provides a details of the nonlinear HRV methods with their theoretical cores for studying the nonlinear behavior of HRV and the findings related to nonlinear HRV analysis under meditative intervention. "Discussion" section provides an overall summary of different approaches. The conclusions and a few future research directions are discussed in "Conclusion and future research trends" section. Finally, "Methods" section provides a detailed search strategy for the selection of relevant research articles analyzed in this study.

## Results

### Distribution of research articles on nonlinear HRV analysis in the last two decades

**Fig. 2:**
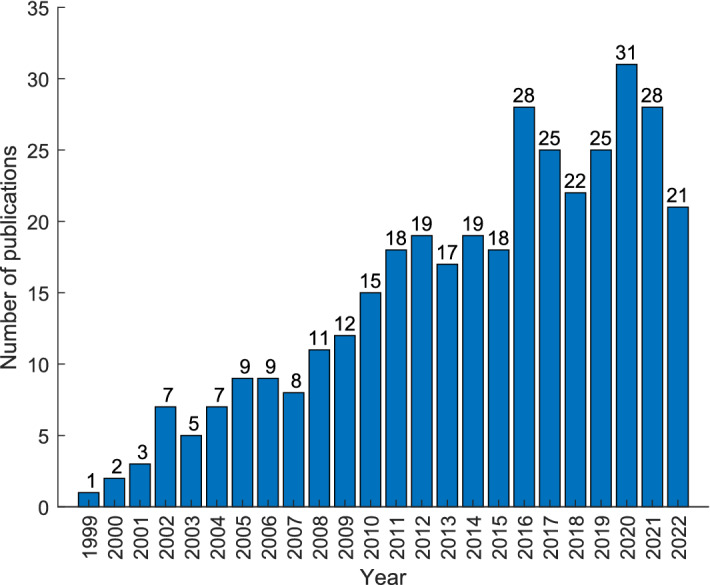
A trend of publications related to HRV analysis for meditation

**Fig. 3:**
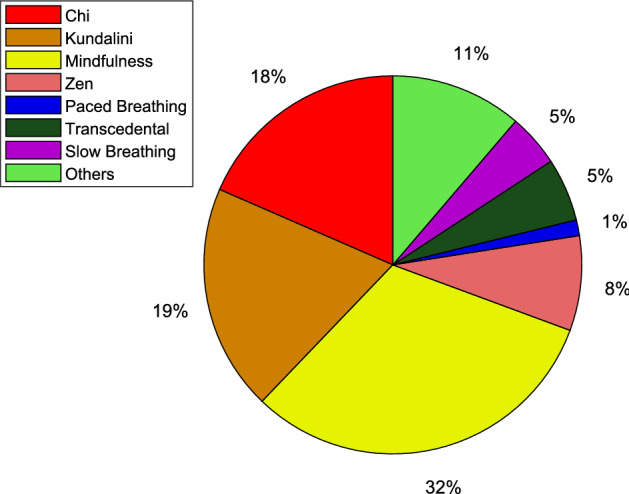
Pie diagram representation of the distribution of meditation techniques used in research articles

The literature search returns around 389-odd articles on nonlinear HRV analysis for studying the cardiac conditions during meditation/yoga. Figure 2 shows year-wise numbers of publications on HRV studies on the above context, which clearly indicates that it is one of the highly researched areas by the scientific community and the trend is fairly on a rising scale. It is worth mentioning that in this study, we have considered Chi, Kundalini, mindfulness, Zen, slow/deep breathing, paced breathing, focused meditation as meditative interventions although there are copious forms of meditation. This is because our aim is to study various nonlinear methods useful for studying meditative interventions rather than to evaluate different meditative interventions. A pie diagram representation of the distribution of meditation techniques used in research articles is shown in Fig. 3.

### Nonlinear methods for studying HRV dynamics

A brief account of the various nonlinear methods used for the study of HRV under meditative interventions; the databases, nonlinear parameters, and significant observations construed from them are detailed in Table 1.

**Table 1:** HRV analysis under meditative condition

Sl. no.	Authors (Year)	Database (subjects)	Nonlinear parameters	Observations/findings during meditation
1	Sarkar and Barat [10] (2008)	PhysioNet (8)	DFA, DEA, Recurrence and MSE analysis	DFA shows strong affect in long-range correlation, DEA exhibits regular repetitive oscillations of time series
2	Papasimakis and Pallikari [32] (2009)	PhysioNet (8)	DFA scale exponent and ShEn	DFA scale exponent decreases; decimated long-range correlation, standard deviation of ShEn decreases at higher scales indicating reduced variations in the correlations of HRV
3	Goswami et al. [13] (2010)	PhysioNet (12) and own (3)	Normalized ShEn	Normalized ShEn decreases for advanced meditators which indicates lower HRV dynamics. No statistical test found
4	Diosdado et al. [29] (2010)	PhysioNet (46)	Higuchi’s fractal dimension (HFD)	HFD graph possesses quasi-periodic components indicating reduced complexity. No statistical test found
5	Li et al. [26] (2011)	PhysioNet (26)	Base-scale entropy (BSEn)	BSEn* decreases, $\pi$-type probability distribution shows more certainty; indicates low complexity of HRV
6	Goswami et al. [33] (2011)	PhysioNet (12), own (3)	Second order difference plot (SODP)	Cluster formed by SODP rotates anticlockwise during meditation; indicates detachment from the external world. No statistical test found
7	Raghavendra and Dutt [24] (2011)	PhysioNet (12)	MED, CD, LLE and NLS	MED* and CD* decrease, whereas LLE* and NLS* increase; inducement of overwhelming calmness and significant alertness
8	Raghavendra and Dutt [12] (2011)	PhysioNet (12)	Fractal dimension	Significantly low fractal dimension*; increases for scales 1 to 7 and then becomes constant
9	Song et al. [12] (2013)	PhysioNet (8)	Multifractal detrended fluctuation analysis, singularity spectrum width	Significantly narrow singularity spectrum width indicating reduced dynamical complexity. No statistical test
10	Jiang et al. [30] (2013)	PhysioNet (12)	Visibility Graph method and *P*(*k*)	*P*(*k*) initially decreases ($k\le 8$) and then significantly increases ($k>$11). Long-range correlation is retained only at higher scales. No statistical test found
11	Goshvarpour and Goshvarpour [34] (2013)	PhysioNet (12)	Higher order spectral (HOS) analysis: Bispectrum estimation	Bispectrum amplitude increases during KYM and decreases significantly ($p<0.05$) during Chi meditation
12	Kamath [15] (2013)	PhysioNet (12)	CCTM and HFD	Significant increase in CCTM; indicates activation of PNS.
13	Goshvarpour and Goshvarpour [35] (2015)	PhysioNet (8)	SD1 (minor axis), SD2 (major axis), area under Poincaré plot	SD1/SD2* increase significantly; elliptical Poincaré becomes circular; indicates definite change in the psychological state
14	Bhaduri and Ghosh [28] (2017)	PhysioNet (12)	Multifractal-DFA and PSVG analysis	PSVG increases during Kundalini yoga and Chi meditation, indicates increase in the degree of complexity. No statistical test found
15	Alvarez-Ramirez [36] (2017)	PhysioNet (12)	Hurst exponent	Hurst exponent decreases; indicating uncorrelated HRV dynamics and destruction of long-range correlation. No statistical test found.
16	Goshvarpour and Goshvarpour [37] (2018)	PhysioNet (12)	Correlation entropy and Cauchy–Schwarz divergence	Correlation entropy* is the lowest and Cauchy–Schwarz divergence* is the highest (low SNS activity)
17	Yao et al. [38] (2018)	PhysioNet (26)	Entropy measures: KW, BS, PEn, and DSJE	All the entropies are significantly lesser. Lower dynamical complexity
18	Guo et al. [39] (2019)	Author’s own (70)	DFA scale exponents $\alpha _1$ and $\alpha _2$	Significant increase in $\alpha _1$ and $\alpha _2$*. Prevalent SNS activity is observed
19	Nasrolahzadeh et al. [40] (2019)	PhysioNet (8)	Graph index complexity (GIC) based on visibility graph	GIC values are significantly higher indicating higher complexity
20	Goshvarpour and Goshvarpour [27] (2019)	PhysioNet (12)	SD1, SD2, LZ complexity, LLE, SampEn, ShEn, ApEn, LogEn	SD1*** and SD2*** show large variations, LLE*** increases, LogEn*** increase but LZ*** complexity, SampEn***, ApEn***, and Shannon entropy*** decrease; indicates low complexity
21	Deka and Deka [41] (2020)	PhysioNet (12)	IncrEn	Decrease in IncrEn during meditation; however the difference is not statistically significant ($p>0.05$).
22	Goshvarpour and Goshvarpour [42] (2020)	PhysioNet (12)	Heart rate asymmetry (HRA) index	Significant increase in HRA index with the increase in lags of NN intervals
23	Rohila and Sharma [43] (2020)	PhysioNet (8)	Asymmetric spread index (ASI), Porta’s index (PI), Guzik’s index (GI), slope index (SI) and area index (AI)	Significant increase in ASI, PI and GI. Crossover of ASI is observed in some meditators. Overall dominant PNS activity
24	Deka and Deka [14] (2021)	PhysioNet (12)	EMD-based Energy ShEn (eShEn), Kurtosis, Skewness, DFA based short-term scale exponent ($\alpha _1$), multiscale PEn (MPE)	Significant decrease in eShEn***, MPE*** at lower scales (1,2,3,4) and $\alpha _1$***. However with the increase in scales, MPE increases during meditation providing a hint of higher underlying complexity
25	Goshvarpour and Goshvarpour [44] (2022)	PhysioNet (12)	Verhulst map-based measures: area, circumradius, inradius	Significant decrease in area, circumradius, and inradius during Chi meditation and significant increase in area, circumradius, and inradius during KYM
26	Deka and Deka [45] (2022)	PhysioNet (12)	Improved multiscale distribution entropy (ImDistEn)	Significant increase in ImDistEn over higher scales (>5) during Chi and KYM meditation as compared to before meditation

*$p<0.05$, **$p<0.01$, ***$p<0.001$, ApEn: approximate entropy, CCTM: component central tendency measures, CD: correlation dimension, DEA: diffused entropy analysis, DFA: detrended fluctuation analysis, DSJE: double symbolic joint entropy, *k*: degree of VG node, KW: Kurths J. and Wessel N., LLE: largest Lyapunov exponent, LogEn: log energy entropy, LZ: Lempel–Ziv, MED: minimum embedding dimension, NLS: nonlinearity score, *P*(*k*): degree distribution, PSVG: power of scale-freeness in VG, SampEn: sample entropy

Next, we elaborate the theoretical background and the mathematical formulation of some of the widely used techniques belonging to nonlinear regime; highlighting the parameters used for studying the dynamics of HRV as follows.

#### Application of Poincaré plots

**Fig. 4:**
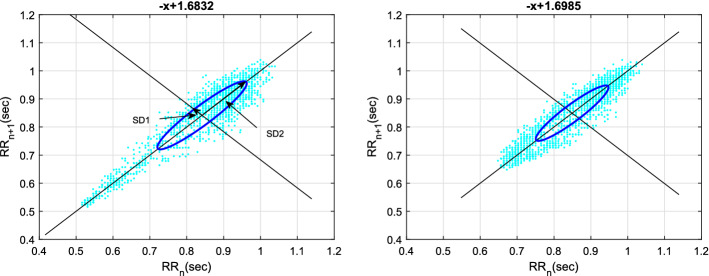
Poincaré plot of HRV signal before meditation (left) and during meditation (right) [42]

Poincaré plot is popularly used to study the behavior of ANS by investigating the short-term and long-term HRV [46, 47]. In this technique, each RR interval is plotted against its previous RR interval (i.e., $RR_{n+1}$ vs. $RR_{n}$ for a delay of 1), which forms a scatter plot as shown in Fig. 4. The shape of the plot gives a qualitative assessment, while the standard deviation parameters, SD1 and SD2 (dispersions along the minor and major axes of the fitted ellipse, respectively) could provide a quantitative assessment of the underlying dynamics of HRV. Particularly, SD1 corresponds to the standard deviation of instantaneous (short-term) variability of RR intervals, which indicates the activity of PNS, while SD2 corresponds to the standard deviation of continuous long-term variability, which is an indicator of the activity of SNS [37, 47]. The SD1/SD2 ratio is the relative measure of short-term and long-term variability of RR intervals.

A new approach of Poincaré analysis is also reported in the form of heart rate asymmetry (HRA) measure by Karmakar et al. [48], and later applied in [42] to assess its sensitivity towards delay. Mathematically, the HRA index, popularly known as Guzik’s index (GI), is given by [48]:

1 $$\text {GI}=\frac{\sum _{i=1}^{C(P_{i}^{+})}(D_{i}^{+})^2}{\sum _{i=1}^{N-1}(D_{i})^2}\times 100\%, $$

where $C(P_{i}^{+})$ is the number of points (cyan-colored dots) above the line of identity (LOI) as shown in Fig. 4, *N* is the number of RR intervals, $D_{i}^{+}$ is the distance of *i*th point above the LOI, and $D_i$ is the distance of *i*th point from the LOI given by:

2 $$D_i=\frac{\left| {\text{RR}(i+1)-\text{RR}(i)} \right| }{\sqrt{2}}. $$

It is reported that HRA index of meditators (Chi and KYM) are significantly different from non-meditators especially at higher delays [42, 43]. Similarly, Rohila and Sharma [43] observe that there occurs a significant increase in GI index too during meditation, while Goshvarpour et al. [27, 35] have observed that SD1/SD2 ratio also increases during meditation, which connotes the dominant parasympathetic activity, while Dey et al. [49] have presented a 3D frequency delay plot, which shows that the effect of Chi and KYM on the ANS are not alike, rather their impacts are completely opposite. Goswami et al. [33] use a modified version of Poincaré plot, termed as the second-order difference plot and observe that during meditation (Chi and KYM), the axis of the cluster rotates anticlockwise. Furthermore, correlation coefficient obtained from consecutive first-order differences of RR intervals also increases during meditation. However, their results are not further validated by statistical significance tests. In order to compare the findings of the standard HRV features along with some of the commonly used nonlinear features (DFA scale exponent and PEn), against the findings of the existing works, we have demonstrated the simulated results in Table 2. It can be observed that only a few of the standard HRV features are able to distinguish the two states of mind–body states significantly. This portrays the importance of nonlinear analysis in distinguishing the HRV data of compared groups having subtle differences.

**Table 2:** Statistical significance test of the HRV features [14]

Parameter	Pre-meditation	During meditation	*p*-value
	Mean ± SD	Mean ± SD	
SDNN (ms)	78.726 ± 39.553	75.334 ± 25.261	0.619
RMSSD (ms)	44.152 ± 24.051	41.292 ± 22.752	0.004*
Kurtosis	3.35 ± 1.33	3.32 ± 1.59	0.804
Skewness	− 0.005 ± 0.278	0.167 ± 0.662	0.135
NN50 count	29.750 ± 19.015	31.260 ± 22.942	0.503
HTI	14.618 ± 4.707	14.315 ± 3.832	0.551
$\alpha _1$	1.078 ± 0.131	.925 ± 0.126	${2.88\times 10^{-15}}$*
DOD	1.214 ± 0.149	1.262 ± 0.161	0.036*
$\text {PEn}$	1.730 ± 0.043	1.5814 ± 0.122	${1.31\times 10^{-22}}$*

* indicates statistically significant values

#### Nonlinear parameters in phase space

*Application of phase space representation* In case of nonlinear analysis of HRV, it is very common to transform the time series into the phase space form. The rationale behind the transformation is to capture all possible dynamical states of the 1-D signal, which would not otherwise be possible from the magnitudes of a single variable. For that purpose, the signal is embedded into higher dimensions. The minimum embedding dimension (MED) provides the minimum number of variables required to represent the complete dynamics. But, in that process it is essential to ensure that the reconstructed attractor of different dimensions preserve the salient properties of the original attractor [24, 50]. The choice of proper embedding dimension and time delay play an important role in the efficient reconstruction of the attractor. Mathematically, the RR interval (HRV) time series of *N* data points, denoted by $\text{RR}(i)=[\text{RR}(1), \text{RR}(2),.....,\text{RR}(N)]$ can be transformed into an *m* dimensional phase space with time delay, $\tau$ by Taken’s embedding theorem [51] as follows:

3 $$ Y=[Y(1), Y(2),.....Y(N-(m-1)\tau ]^T, $$

where each time delay vector, *Y*(*i*) can be expressed as $Y(i)=[\text{RR}(i), \text{RR}(i+\tau ), \text{RR}(i+2\tau ),...,\text{RR}(i+(m-1)\tau )]$ and there are $N_v=N-(m-1)\tau$ such time delay vectors.

**Fig. 5:**
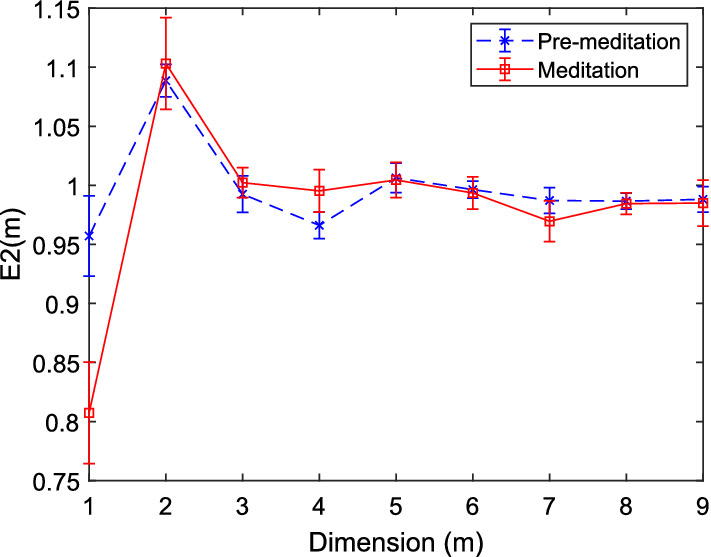
Variation of the E2(m) against embedding dimensions to determine MED for HRV time series corresponding to meditative and pre-meditative states using Cao’s method [50]

**Fig. 6:**
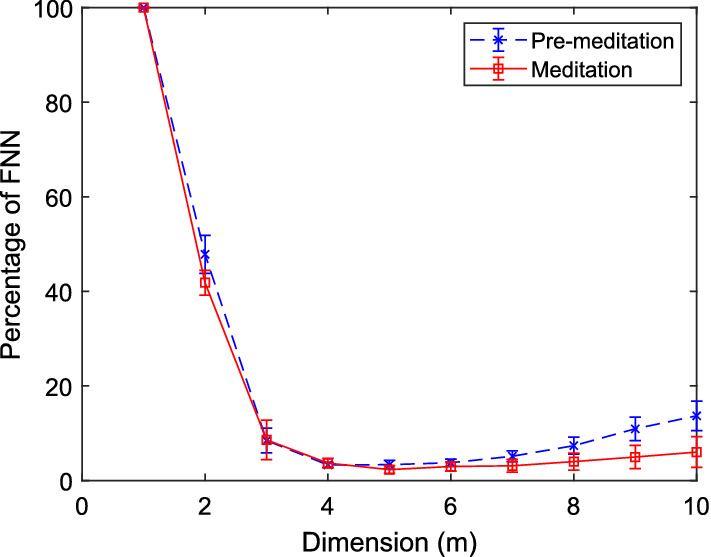
Variation of percentage of FNNs against embedding dimensions to obtain MED for HRV time series corresponding to meditative and pre-meditative states using Kennel’s method [52]

**Fig. 7:**
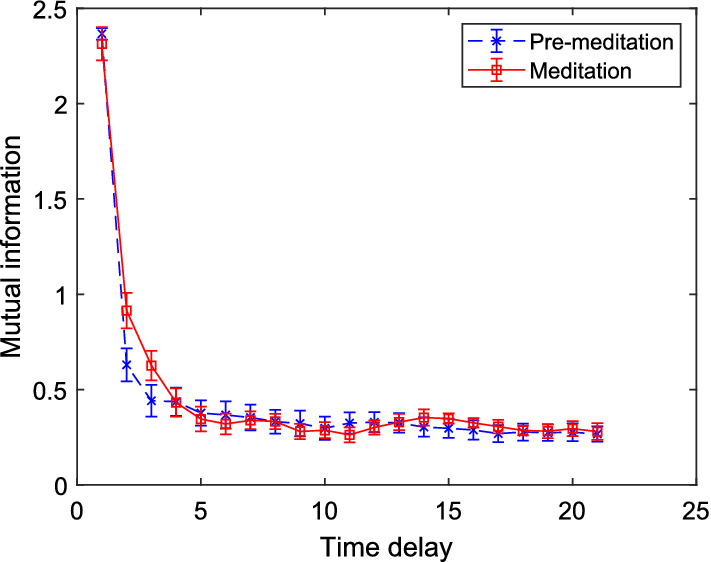
Variation of mutual information against varying time delay for HRV time series corresponding to meditative and pre-meditative states

For the estimation of MED, generally the methods based on the singular value decomposition (SVD) [53], the variation of the exponent of power-law based correlation integral [54], the count of false nearest neighbors (FNNs) based on Kennel’s [52] and Cao’s [50] approaches are commonly used. However, the method [50] has received wide acceptance among the chaos analysts because of the computational ease and reliability. Another advantage is that the estimation of FNNs is threshold independent. The attractor gets unfolded if the signal is embedded at higher dimension than MED, which can facilitate the study of stability, predictability, correlation behavior, and regularity. However, it is to be noted that the optimal time-delay value should be known prior to the estimation of MED. Average mutual information (AMI) and autocorrelation techniques are mostly used to determine the right delay at which these functions return the least value. Figures 5 and 6 show estimation of MED based on Cao’s and Kennel’s methods, respectively, for an HRV signal of meditators (PhysioNet). Here, *E*2(*m*) is given by $\frac{E^*(m+1)}{E^*(m)}$, with $E^*(m+1)$ and $E^*(m)$ indicating the averages of the distances between the neighboring vectors at dimensions $m+1$ and *m*, respectively, as given below:

4 $$ E^*(m)=\frac{1}{N-m\tau }\sum _{i=1}^{N-m\tau } \left| \text{RR}_{i+m\tau }-\text{RR}_{n(i,m)+m\tau } \right| , $$

where $RR_{n(i,m)+m\tau }$ is the nearest neighbor of $RR_{i+m\tau }$ in the *m*-dimensional phase space. As reported in [50], *E*2(*m*) is specially tailored to distinguish deterministic signals from stochastic signals. For the test HRV signal, it attains the peak value at $m=2$ and then saturates beyond $m=3$. Saturation of *E*2(*m*) after $m=3$, gives the value of MED, i.e., $m+1=4$. As per Kennel’s method since the lowest percentage of FNN is found at $m=4$ with threshold of 15% false neighbors, the MED = 4.

It can be observed from Fig. 7 that mutual information drops sharply and it remains negligible from $\tau =4$ onward. With lower $\tau$ values, the MED required to capture the attractor of a signal is higher than that with optimal $\tau$ values. However, time-delay information is more relevant for continuous time signals, while for the analysis of discrete time signal (e.g., HRV), the time-delay of 1 is sufficient [50].

**Fig. 8:**
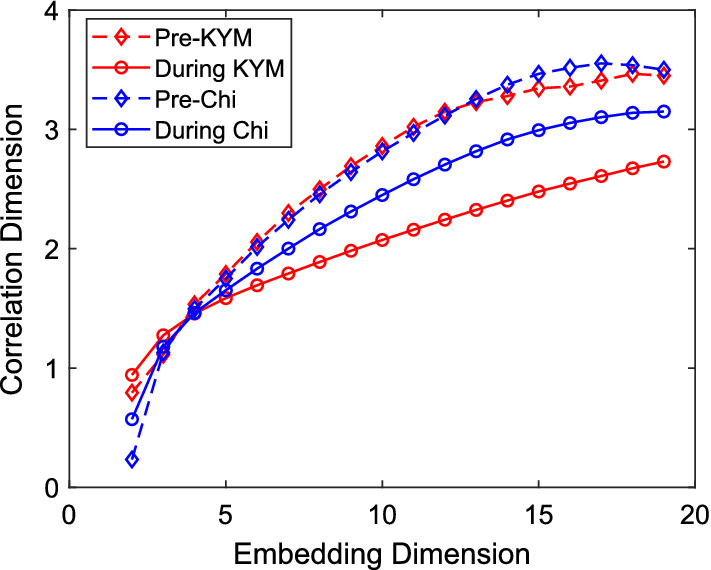
CD values of HRV time series after embedding the series with varying embedding dimensions *m* for meditative and pre-meditative states [24]

Phase-space transformation is found to be the basis for various types of nonlinear analyses, since it provides the scope to analyze a 1-D time series under higher dimensions. This led to its use in studying the effects of meditation from the aspects of asymptotic stability [24, 27, 55], time correlation of phase trajectories [10, 27, 55], dynamical complexity [10, 14, 26, 37, 41, 45], etc. Nevertheless, the computation of MED from phase space representations using FNN and Cao’s techniques (shown above) have revealed that HRV signals under meditative and pre-meditative conditions can be best studied within MED=3. This indicates that even during meditative states, the HRV time series maintains the property of lower order deterministic chaos. Thus a general belief that the meditation may develop more regular and predictive cardiac dynamics is actually false, rather the cardiac dynamics still remain complex. It is worthwhile to mention that a complex heart dynamics indicates better adaptability of heart, whereas the monotonous heart dynamics enunciates pathological heart conditions.

*Application of correlation dimension (CD)* It gives the assessment of the complexity of a time series [54]. Since, for many complex signals, CD value is a fractional quantity, it is also known by fractal dimension (FD). Higher the magnitude, greater is the dynamical complexity. CD-based dimension calculation is having more adoption in biomedical applications as compared to the other techniques including, box-counting dimension, Kolmogorov capacity dimension, and information dimension, since it needs relatively lesser data for its calculation. It can be obtained from a time series by finding correlations sum calculated from its data points as reported in [56]:

5 $$ C_{m}(r)=\frac{1}{N_v}{\sum _{i=1}^{N_v}C_{i}^m(r)}, $$

where

6 $$\begin{aligned} C_{i}^m(r)= {\left\{ \begin{array}{ll} \frac{1}{N_v-1}\sum _{j=1,j\ne {i}}^{N_v}[{\mathcal {H}}(r-Y_{i,j}^m)],&{} \text {if } i\le {N_v}\\ 0, &{} \text {otherwise} \end{array}\right. }, \end{aligned}$$

is the correlation integral of all the correlation functions, $N_v$ is the number of states, and ${\mathcal {H}}$ is the Heaviside step function, which determines whether the distance between *m* dimensional vectors, $Y_{m}(i)$ and $Y_{m}(j)$ is within a sphere of radius, *r* or not. $C_{m}(r)$ is found to have a power-law relationship with *r*, whose exponent gives the measure of CD [54]:

7 $$ \text {CD}=\lim _{r\rightarrow 0}\frac{\log C_{m}(r)}{\log (r)}. $$

Raghavendra and Dutt [24] observed reduced MED and CD values during meditation (Chi and KYM) as shown in Fig. 8, which they considered to be due to a state of overwhelming calmness. Although CD is considered as an indicator of vagal activity, it also characterizes the sympathovagal balance [57, 58]. This demonstrates the depressed vagal activity during meditation. However, it is to be mentioned that estimation of CD requires large number of data, which makes its use dubious for the given dataset having smaller data length.

**Fig. 9:**
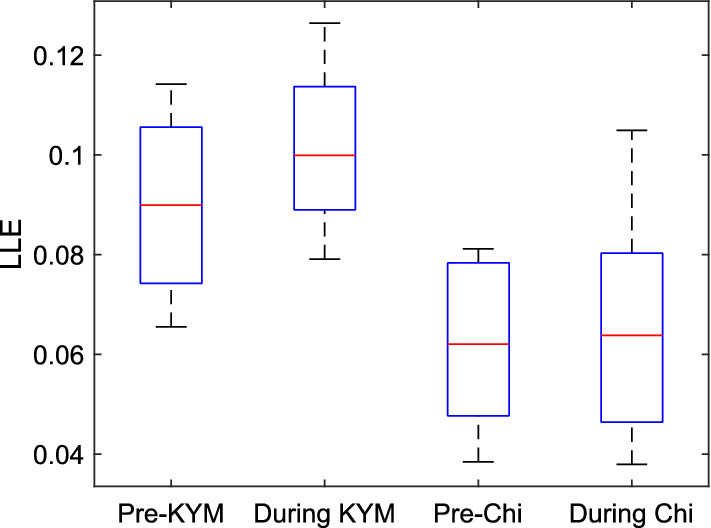
LLE for HRV time series corresponding to meditative and pre-meditative states [24]

*Application of Lyapunov exponent (LE)* LEs indicate whether the underlying system of a time series is chaotic or not [59]. It determines the sensitivity of a system to the initial conditions by calculating the average rate of separation of infinitesimally close trajectories. A positive value of the largest LE (LLE) of a series means that the underlying system is chaotic, while negative LE describes the asymptotic stability (convergence of close trajectories). For a phase space representation of *m* dimensional dynamical series having *N* points, let the spectrum of LEs be $(\lambda _{1},\lambda _{2},...\lambda _{m})$. If the rate of divergence between any two trajectories is $\delta Z(t)$ with initial separation vector $\delta Z_{0}$, the LLE at a time *t* can be obtained by [24]:

8 $$ \text {LLE}=\lim _{t\rightarrow \infty }\lim _{\left| \delta Z_{0} \right| \rightarrow 0}\frac{1}{t}\frac{\left| \delta Z(t) \right| }{\left| \delta Z_{0} \right| }. $$

Fig. 9 shows that LLE is higher during meditation [24, 27, 55], which is considered to be associated with enhanced alertness during meditation. The higher value of LLE during meditation may be considered as the manifestation of stronger neurocardiovascular couplings as well as the couplings between the ANS and other sensory systems [60, 61].

*Application of recurrence plot and recurrence quantification analysis* The recurrence plot (RP) is an array of dots, graphically represented by a square-sized ($N \times N$) image. A dot is placed at (*i*, *j*) whenever the delay vector $Y_m( j)$ is sufficiently close to another vector $Y_m(i)$. Therefore, the index (*i*, *j*) corresponds to the time information, and RP naturally describes the time correlation between two vectors. Its not restrained by stationarity, dimension size, and data length. Mathematically, the vectors, $Y_m(i)$ and $Y_m(j)$ are recurrent if they are within a cut-off distance ($\varepsilon$) [10, 62, 63], i.e.:

9 $$ R_{i,j} ={\mathcal {H}}(\varepsilon - \left\| Y_m(i)-Y_m(j) \right\| ). $$

**Fig. 10:**
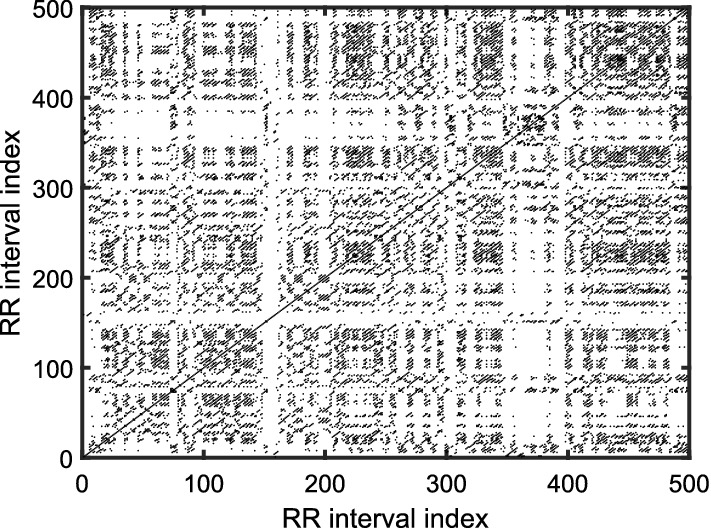
Recurrence plot of representative HRV time series before meditation [10]

**Fig. 11:**
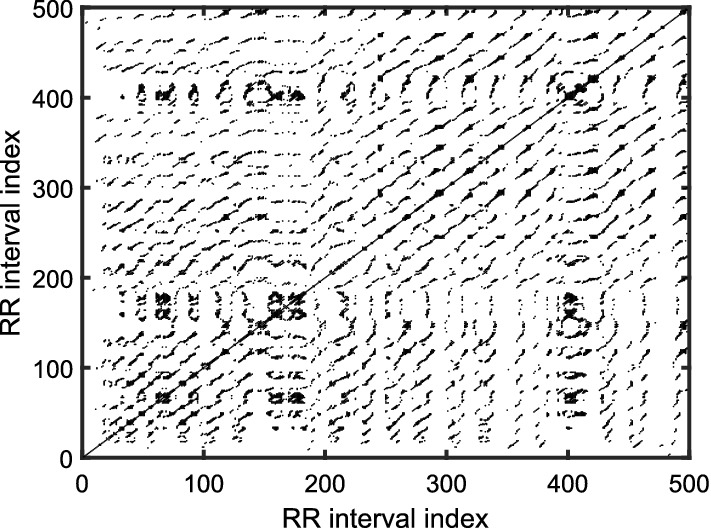
Recurrence plot of representative HRV time series during meditation [10]

For clarity, recurrence plots for HRV signal corresponding to meditative and pre-meditative states are presented in Figs. 10 and 11 under a setting of embedding dimension, *m*=3 and time delay=1. It can be seen that during meditation, more prominent short lines parallel to the diagonal of the RP are found. This indicates that some states recur during meditation hinting a more periodic kind of oscillations. These periodic patterns may be due to the controlled breathing cycles during meditation. This illustrates the importance of respiratory data while analyzing the effects of meditation to arrive at a more profound decision. On the other hand, it is equally important to understand the subtle patterns in the RP, which may not be ascertained by visual inspection. Nonetheless, the correlations between different sub-systems can be better analyzed by mathematical tools, such as recurrence quantification analysis (RQA) using the recurrence rate (REC), the determinism (DET), the maximal length of diagonal structures (${L}_{\text{max}}$), the Shannon entropy, and the trend based on the RP [63]. Later on, Marwan et al. [64] have derived few more quantifiers namely, laminarity, trapping time, and maximal length of vertical structures to extract further information from RP.

Goshvarpour and Goshvarpour [27, 55] observe that big black rectangular structures in the RP change to small rectangular patches when subjects sink in the meditative state from pre-meditative state. Sarkar and Barat [10] have observed that RQA parameters remain nearly constant at varying time scales during Chi meditation, whereas the parameters vary abruptly before meditation. On a nutshell, the information about the temporal correlations over certain time intervals and the information about the impact of RSA on baroreceptor sensitivity as well as the autonomic activity can be strongly analyzed by RP tools.

*Application of entropy in phase space* Entropy-based parameters are also computed in phase space to reveal the regularity, complexity, and predictability of a system [26, 65, 66]. A brief overview of a few of them are given below:

*Approximate entropy (ApEn)* It provides the degree of regularity and unpredictability of the variations in a time series [67], which is given by:

10 $$ \text {ApEn}=\phi ^{m}(r)-\phi ^{m+1}(r), $$

where

11 $$ \phi ^{m}(r)=(N-m+1)^{-1}\sum _{i=1}^{N-m+1}\log [D_{i}^{m}(r)]. $$

Here, $D_{i}^m(r)$ is almost the same as $C_{i}^m(r)$ of Eq. 6, differing only for its consideration of distance measurement of time delay vectors with itself; $\phi ^{m}(r)$ is the average of the correlation functions of all the time delay vectors with dimension *m*, and *r* is the tolerance limit.

*Sample entropy (SampEn)* Since approximate entropy is dependent on the data length and includes self matching of time delay vectors, it can give rise to biased result [67]. So it has been modified to derive the sample entropy (SampEn), which provides better realization of complexity of a time series. Its mathematical expression is given by [68]

12 $$ \text {SampEn}=-\log \frac{A}{B}, $$

where *A* and *B* are the number of time delay vectors whose Chebyshev distances are less than *r* in $m+1$ and *m* dimensional phase space, respectively.

*Permutation entropy (PEn)* This entropy is suitable for identifying whether a temporal dynamics is originated from stochastic process or deterministic chaos, and follows the identical mathematical formulation as used in ShEn [66]. After embedding the time series into phase space, the unique ordinal patterns of the embedding vectors are designated as $\pi$ forms. The maximum possible patterns configurable by the vectors of *m* dimension is given by *m*!. It is mathematical expression is given by:

13 $$ \text {PEn}=-\sum _{i=1}^{m!}P(\pi _i)\log _{2}P(\pi _i). $$

*Base-scale entropy (BSEn)* BSEn is another complexity metric suitable for short time series. For its computation, firstly the base scale (BS) is obtained from the phase space representation of the series and then symbolic sequences are formed on the basis of BS. Its mathematical calculation is given by [69]:

14 $$ \text {BS}=\sqrt{\frac{\sum _{k=1}^{n}[Y(i+k)-Y(i+k-1)]^2}{m-1}}. $$

The symbolic sequences are coded in terms of symbols (say, a = 0, 1, 2, 3), which indicates about the amplitude resolution. In this case, for *m* dimensional phase space, there can be maximum of $4^m$ (i.e., $\{{\text{length(a)}}\}^m$) different forms of $\pi$. For each $\pi$, the probability of occurrence is calculated to obtain BSE as given below:

15 $$ \text {BSEn}=-\sum _{i=1}^{n}[P(\pi )\log _{2}P(\pi )]. $$

*Increment entropy (IncrEn)* IncrEn evaluates the dynamical complexity of a time series by encoding each increment of it onto a word of two letters, where the first letter denotes the sign of change and the second letter denotes the amount of change [70]. Let these codewords be denoted by $w_{n,k}$ for *n*=1, 2,...,$(N-m)$ and *k* = 1,..,*m* for an embedded increment series, HRV of length *N*; firstly, the successive differences are obtained and then the resultant increment series is embedded into *m* dimensional vectors, $y_n$ for *n* = 1, 2,...,$(N-m)$. Each element of these vectors ($y_n$) are mapped onto a word $w_{n,k}$ for *k* = 1,..,*m* having two letters, say $s_{n,k}$ and $q_{n,k}$ corresponding to sign and symbolic increment, respectively. These *m* words ($w_{n,k}$) in a vector, form a full word denoted by $w_n$ [41]:

16 $$\begin{aligned} q_{n,k}= {\left\{ \begin{array}{ll} 0,&{} \text {if } std(y_n)= 0\\ min(r,\frac{\left\| y_n\right\| \times r }{std(y_{n})}), &{} \text {otherwise} \end{array}\right. } \end{aligned}$$

If $t(w_{n})$ denotes the number of occurrences of *n*th unique word, the probability of occurrence of each unique word is given by:

17 $$ P(w_{n})=\frac{t(w_{n})}{N-m}. $$

The increment entropy, *H*(*m*) for a resolution level (*r*) is given by:

18 $$ H(m)=\sum _{n=1}^{(2r+1)^m}P(w_n)\log _{2}P(w_n). $$

Deka and Deka [41] have observed reduced IncrEn value especially during KYM and marginally lower entropy during Chi meditation than that before meditation, though the results are not statistically significant. This indicates that there may be a reduced irregularity and dynamical complexity during meditation. However, many other markers have demonstrated more significant decrease in entropy values during meditation. For example, BSEn [26], SampEn and ApEn [27], PEn [38] return significantly lower value ($p<0.05$) for the HRV during meditation. However, based on multiscale entropy (MSE) analysis as introduced by Costa et al. [65], Sarkar and Barat [10] show that for higher scales, the SampEn values are quite higher during meditation. In [14], Deka and Deka have used multiscale PEn (MPE) to examine the dynamical complexity of temporal structures in HRV signals of meditators and observed consistent results with that of [10]. Consistent results are also obtained from a recently developed improved multiscale distribution entropy measure [45]. It provides an interesting fact that actually complexity of HRV signal increases during meditation.

Apart from these entropy markers based on Shannon entropy theory, generalized forms of entropies, such as Renyi entropy, Tsallis entropy, Kolmogorov–Sinai (KS) entropy as well as correlation measure-based correlation entropy are also used in practice.

*Renyi entropy (ReEn)* ReEn is the generalized form of ShEn, which is suitable for quantifying the complexity of multifractal time series. The mathematical formulation of ReEn of order *q* can be given as:

19 $$ \text {ReEn}=\frac{1}{1-q}\log \left(\sum _{i=1}^{n}P_{i}^{q}\right), $$

where n is the number of possible outcomes of a signal/series and $q\ge 0$, but $q\ne 1$.

*Tsallis entropy (TsallisEn)* TsallisEn is another generalized form of ShEn, which was introduced with the aim to provide a better analysis of especially, nonextensive systems, very common in natural phenomena. For an order *q*, TsallisEn can be given by [71]:

20 $$ \text {TsallisEn}=\frac{1}{q-1}\left(1-\sum _{i=1}^{n}P_{i}^{q}\right). $$

*Kolmogorov–Sinai (KS) entropy* KS entropy also quantifies the dynamic behavior of a time series, by determining the changes in entropy by partitioning the data at each iteration. To obtain KS entropy, the HRV time series is first mapped using measure-preserving transformation, $T:\text{RR}\rightarrow {\text{RR}}$. The transformed RR series is then partitioned into several parts and each partition elements are considered as intervals $I_i$. Then with the elements in partition at time *n* falling into an interval $I_{i_1}$, while elements falling into interval $I_{i_2}$ at time $n+1$ and so on, block entropies of block size *m* can be obtained using the joint probability $P_{i_1,i_2,..,i_m}$ function by [59]:

21 $$ H_q(m,P_\epsilon )=\frac{1}{1-q}\ln \left(\sum _{i_1,i_2,..,i_m}P_{i_1,i_2,..,i_m}^{q}\right), $$

where $h_q=\underset{P}{\sup }\ \underset{m\rightarrow \infty }{\lim }\frac{1}{m}[H_q(m,P_\epsilon )]$ and $\underset{P}{\sup }$ means finding the supremum over all possible *P*, provided $\epsilon \rightarrow 0$.

*Correlation entropy (CorrEn)* It gives the similarity measure between two random variables in the neighborhood of joint space. For this, the data are transformed into a higher dimensional Hilbert space and afterwards the correlation of the data in Hilbert space is obtained. If *X*, *Y* are two series of same dimension then CorrEn can be obtained by [37]:

22 $$ \text {CorrEn}= E[<H(X),H(Y)> ]=E[K_{\sigma }(X,Y)], $$

where *H*(*X*) and *H*(*Y*) map the data *X* and *Y* to higher dimensional Hilbert domain. $K_{\sigma }$ is a Kernel function, which could be a polynomial function, radial basis function (RBF) or sigmoid function, etc.

**Fig. 12:**
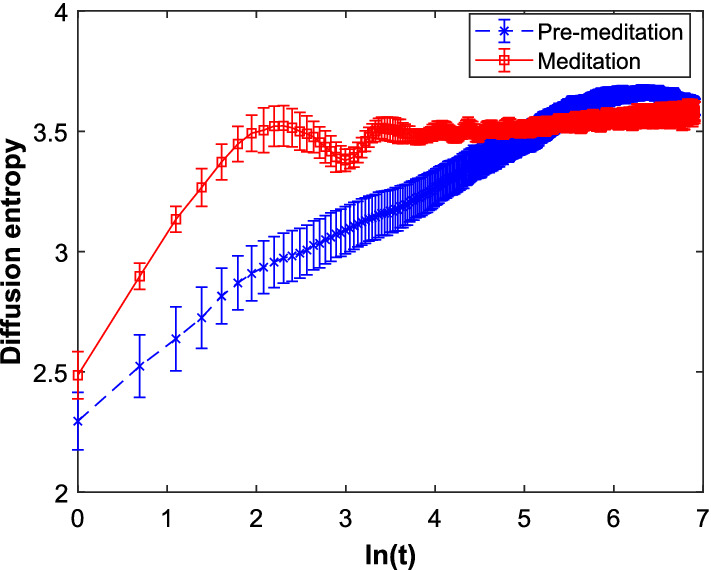
Error bar plot of DEA for Chi meditators’ HRV time series during and before meditation [10, Fig. 3]

Goshvarpour and Goshvarpour [37] evaluate information theoretic descriptors, namely, the CorrEn and the Cauchy–Schwarz divergence (CSD) and observe very low CorrEn values during Kundalini yoga for smaller kernel sizes. It indicates higher relaxed state and lower SNS tone during Kundalini yoga meditation. However, as the kernel size increases, these values further decrease marginally. They further conclude that CSD fails to demonstrate the nonlinear similarities. Again, diffusion entropy analysis (DEA) as shown in Fig. 12 demonstrates regular repetitive oscillations during meditation, indicating the loss of long-range correlation. In [72], Porto et al. have found that although entropy markers, such as TsallisEn, ReEn could not provide significant difference between slow breathing and normal breathing conditions. They observed that dynamical complexity in case of slow breathing reduces and this leads to an reduced vagal control.

#### Fractal/multi-fractal analysis

*Applications of detrended fluctuation technique* Like many natural objects and signals, such as mountains, leaves of plants, DNA sequences, time series of network traffic, HRV time series also possesses the fractal behavior. Fractal object/process should have a fractional dimension and possess self-similarity behavior under different spatial/temporal scales. Fractal dimension (FD) is the most fundamental measure to check the fractality and hence the complexity of a signal, which is based on the degree of space-filling of a curve constituting the signal. Even though CD, box-counting dimension, Hausdorff dimension can provide the measure of FD, Hurst exponent and detrended fluctuation analysis (DFA) based FD measures are widely used [32, 73].

**Fig. 13:**
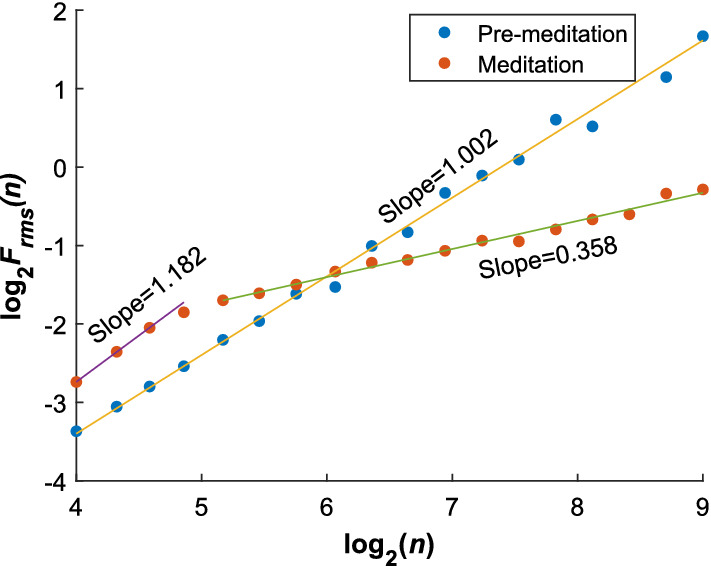
DFA of representative instantaneous HRV time series during and before meditation [10, Fig. 3]

DFA method reveals the possession of self-similarity (fractal) behavior by eliminating any kind of trends. This trend unless removed, could mislead the interpretation of fractal analysis as it could add unnecessary signal components extraneous to the underlying dynamics of interest. The mathematical formulation of this method is given below [73]:

23 $$\begin{aligned} &F_{rms}(n)=\sqrt{\frac{1}{N}\sum _{m=1}^{N}[\text{RR}_{cum}(m)-\text{RR}_{t_{n}}(m)]^2},\\&F_{rms}(n)=n^{\alpha }, \end{aligned} $$

where $\text{RR}_{cum}(m)=\sum _{i=1}^{m}[RR(i)-\overline{RR(i)}]$, $\overline{\text{RR}(i)}$ is the mean of RR interval series, $\text{RR}_{cum}(m)$ is the cumulative sum, $F_{rms}(n)$ is the rms fluctuation of the series at scale *n*, $\text{RR}_{t_{n}}(m)$ is the trend (least-square fit line) of RR(*m*) at scale *n*, and $\alpha$ is the scaling exponent which defines the self-similarity behavior of a signal. For $0\le \alpha \le 1$, Hurst exponent, *H* = $\alpha$ and for $1<\alpha \le 2$, *H* = $\alpha -1$. Here, *H* value reflects the correlation behavior of a series with 0 < *H* < 0.5 indicating antisymmetric correlation and 0.5 < *H* < 1 indicating long-range correlation. From the *H* value, FD can be obtained by: FD = 2-H [74, 75]. It is to be noted that for the computation of asymptotic $\alpha$, a very long time series is required (approximately 9000 data points), which is often unavailable as the physiological signals are concerned. This led to its evaluation for short time scales (4–16 data points), by short-term DFA exponent ($\alpha _s$), which is also found to be effective in studying self-similarity property [27, 73]. In a work, Sarkar and Barat [10] observe that the long-range correlation of the HRV data is destroyed during Chi meditation as shown in Fig. 13, where there is a scaling crossover indicating the effect of meditation. In [12], Raghavendra and Dutt compute the mean and standard deviation of FD values for various scales (from 1 to 20) and it is found that FD values are significantly lesser during meditation ( $p<$0.05). During deep meditation, Guo et al. [39] have found that both short-term and long-term scaling exponents have significantly increased. In another work, Kamath [15] observes decrement in HFD, which is interpreted as the increase of LFp owing to the shift of the RSA by parasympathetic mediation. Alvarez-Ramirez et al. [36] have observed a decrement in Hurst exponent (HE) during chi and KYM meditation from the rescaled range (R/S) analysis. From the time series, firstly overall rescaled range (R/S) is obtained. The constant of proportionality between the logarithm of R/S value and the logarithm of time scales gives the exponent known as HE. Reduced HE indicates uncorrelated HRV dynamics and destruction of long-range correlation due to meditation. In another study, Gronwald et al. [76] have observed that a reduced short-term DFA exponent manifests the reduced dynamical complexity and withdrawal of organismic system to maintain homeostasis under the influence of central nervous system.

Moving forward, it was observed with time that HRV time series do not have the same fractal behavior all throughout its length, rather they possess multifractal behavior. In multifractal analysis method, the fractal behavior is analyzed under different scales locally. More mathematical details about MFDFA can be found in [77]. It is observed by Song et al. [78] that during Chi meditation, multifractal spectrum width reduces as compared to that in pre-meditative state. This indicates a reduced multifractality during meditation.

**Fig. 14:**
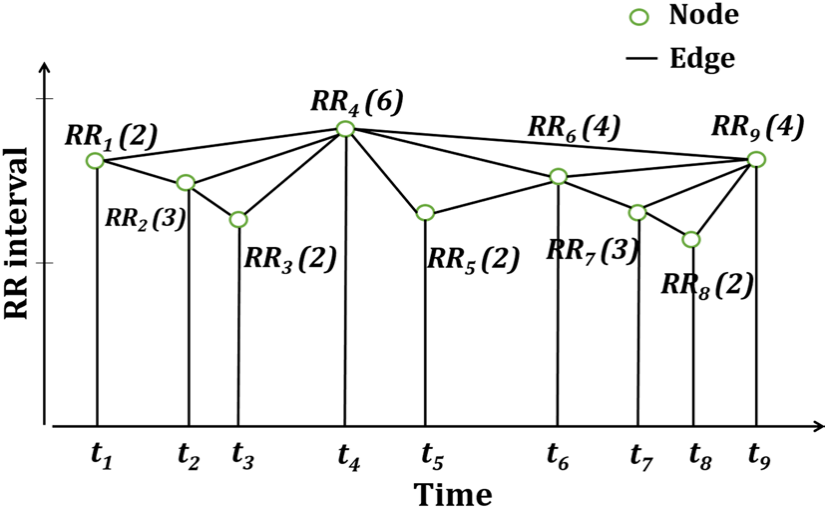
Representation of visibility graph of an HRV time series

*Application of visibility graph technique* Visibility graph (VG) essentially converts a fractal time series into a scale-free graph. In this method, each data points are indicated by nodes and the nodes are connected by edges as shown in Fig. 14. If $X_{p}$ and $X_{q}$ are two data points at positions *p* and *q*, respectively, in a time series then they can be connected by an edge if it satisfies-

24 $$ X_{p+s}< X_{q}+\frac{q-(p+s)}{q-p}(X_{p}-X_{q}), $$

where *p*, *q* and $s\in Z^+$ and $p<p+s<q$. The degree of a node is the number of edges connected with a node, and the degree distribution, *P*(*k*) is the fraction of the nodes with degree *k*. It is to be noted that as per the law of visibility graph, degree distribution follows power-law with the degree of a node, i.e., $P(k) =k^{\lambda _{p}}$. The constant of proportionality, $\lambda _{p}$ is called power of scale-freeness in visibility graph (PSVG) [28].

Based on VG, several complexity markers were derived, namely, medium articulation ($\text {MA}_{VG}$), graph index complexity (GIC), Visibility entropy (VGEn), offdiagonal complexity (ODC), etc. [79]. It was found that graph with medium number of edges is the most complex, whereas both sparse type and fully connected graphs are in fact less complex. $\text {MA}_{VG}$ value attains the maximum value for such graphs with medium edges, which follows $n_{VG}^{1.5}$, with $n_{VG}$ being the number of nodes. Again, ODC provides the diversity in the value of node–node edges. More mathematical details about $\text {MA}_{VG}$ and ODC can be found in [79]. However, since GIC and VGEn have been more frequently used in time series analysis including HRV, we provide below a brief mathematical details of them:

25 $$ \text {VGEn}=-\sum _{k=1}^{D}P(k)\log _{2}P(k), $$

where *D* is the total number of unique degrees. On the other hand, GIC is determined from the eigenvalues of adjacency matrix of a graph. The largest eigenvalue ($\lambda _{max}$) for a graph lies with the range [$2\cos (\frac{\pi }{n_{VG}+1}), (n_{VG}-1)$]. Mathematically, GIC is given by [40, 79]:

26 $$ \text {GIC}=4c(c-1), $$

where $$c=\frac{\lambda _{max}-2\cos (\frac{\pi }{n_{VG}+1})}{n_{VG}-1-2\cos (\frac{\pi }{n_{VG}+1})}.$$

**Fig. 15:**
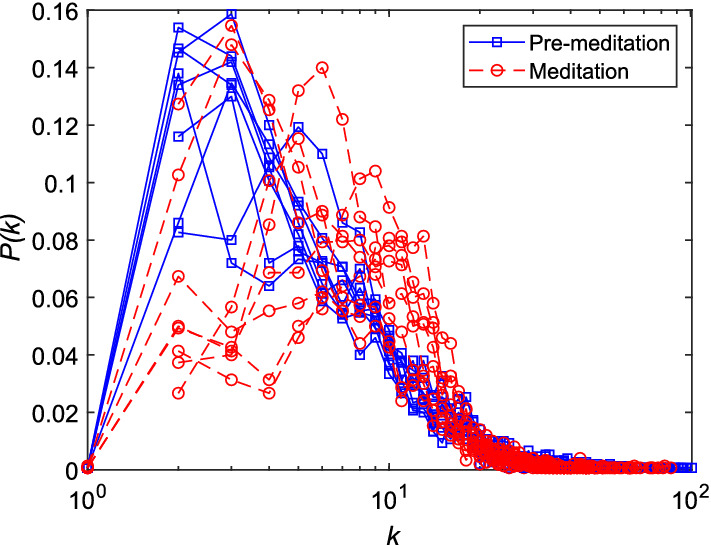
The degree distribution, *P*(*k*) of visibility graph network for HRV time series of meditators [30]

The PSVG depicts the amount of complexity and self-similarity of a dynamic time series, which is also linearly related to the HE. Higher the PSVG, more is the complexity. Bhaduri et al. [28] observe increase in both multifractal spectrum width and PSVG during Chi meditation. Unlike MFDFA technique, PSVG can be applied for small data size also. However their results contradict to that of [12, 29, 78]. On the other hand, Jiang et al. [30] observe that *P*(*k*) decreases as the degree of node *k* increases before meditation as shown in Fig. 15. Although during meditation it is found to be small for $k<$8, increases consistently to reach the peak value at $k=$11 indicating distinct change in dynamics. However, at large values of *k*, the *P*(*k*) distribution follows a power-law tail, which indicates that the long-range correlation of HRV time series is similar for both pre-meditative and meditative states. Again, from the complexity measure GIC, Nasrolahzadeh et al. [40] have found that during meditation, GIC values are much higher than that before meditation. They corroborate that the higher complexity associated with meditative state, reflects better adaptability of heart under different conditions (i.e., healthy ANS activity).

*Application of higher order spectral (HOS) analysis* HOS analysis provides the spectral analysis of higher order ($>2$) moments or cumulants of a signal. It is very effective in extracting hidden information from nonlinear and non-Gaussian signal (e.g., HRV), which cannot be obtained from regular power spectrum. This is because regular power spectrum provides no information about the Gaussianity/non-Gaussianity behavior of a signal. Besides, power spectrum (second-order moment) is inadequate to provide the phase relations among spectral components [80–82].

If *X*(*k*) is an HRV time series, the *n*th-order spectrum $S_n(f_1,f_2,...,f_{n-1})$ can be obtained by taking the Fourier transform of the *n*th-order cumulant, $c_n(t_1,t_2,...,t_{n-1})$ as given below:

27 $$\begin{aligned} \begin{aligned} S_n(f_1,f_2,..,f_{n-1}) ={}&\sum _{t_1=-\infty }^{\infty }...\sum _{t_{n-1}=-\infty }^{\infty }\{c_n(t_1,t_2,..,t_{n-1}) \\&. \exp [-j\sum _{i=1}^{n-1}2\pi f_it_i]\}. \end{aligned} \end{aligned}$$

The spectrum obtained from 3rd-order cumulant, known by bispectrum is very popular among researchers to capture new information from HRV signal [34, 81–83]. The mathematical expression of bispectrum is given by:

28 $$ S_3(f_1,f_2)= E[X(f_1)X(f_2)X^*(f_1+f_2)], $$

where $E[\cdot ]$ is the expectation operator, *X*(*f*) represents the Fourier transform of a signal *X*(*k*), and $*$ indicates the complex conjugate operation. It is evident from Eq. (28) that bispectrum is capable of providing information about the phase coupling or spectral interactions of a signal at frequencies $f_1$, $f_2$, and $f_1+f_2$. Similarly, trispectrum of a signal can provide details of the spectral interactions of $f_1$, $f_2$, $f_3$, and $f_1+f_2+f_3$ and given by:

29 $$ S_4(f_1,f_2,f_3)= E[X(f_1)X(f_2)X(f_3)X^*(f_1+f_2+f_3)]. $$

In order to eliminate the impact of the variability of power spectrum across different signals on the salient phase coupling information, often bispectrum and trispectrum are normalized. The normalized versions of these two polyspectra are known by bicoherence and tricoherence, respectively. Their mathematical expressions are given by:

30 $$ B_{coh}(f_1,f_2)= \frac{E[X(f_1)X(f_2)X^*(f_1+f_2)]}{\sqrt{E[\left| X(f_1)X(f_2) \right| ^2]E[\left| X(f_1+f_2) \right| ^2]}}, $$

31 $$ {T_{coh}(f_1,f_2)= \frac{E[X(f_1)X(f_2)X(f_3)X^*(f_1+f_2+f_3)]}{\sqrt{E[\left| X(f_1)X(f_2)X(f_3) \right| ^2]E[\left| X(f_1+f_2+f_3) \right| ^2]}}}. $$

**Fig. 16:**
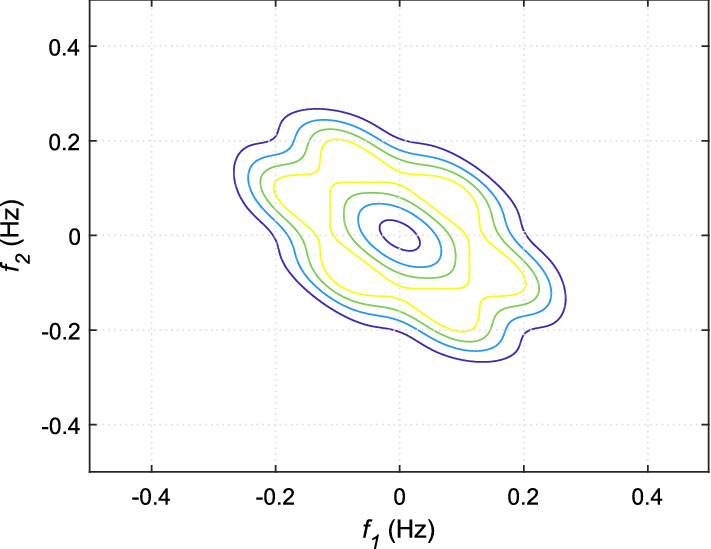
Bispectrum plot for HRV signal during pre-meditative state [34]

**Fig. 17:**
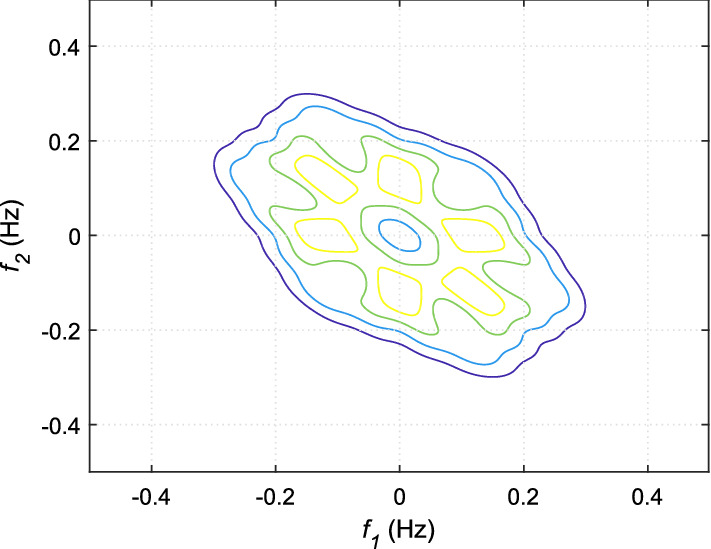
Bispectrum plot for HRV signal during meditative state [34]

**Fig. 18:**
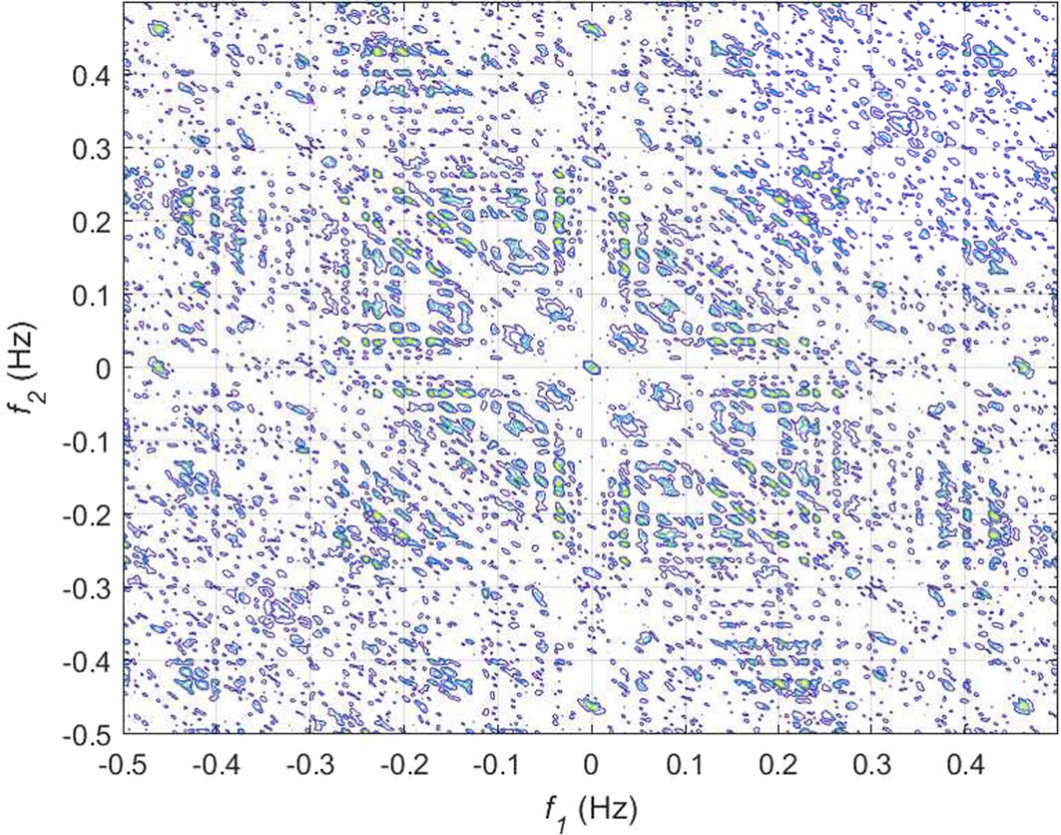
Bicoherence plot for HRV signal during pre-meditative state

**Fig. 19:**
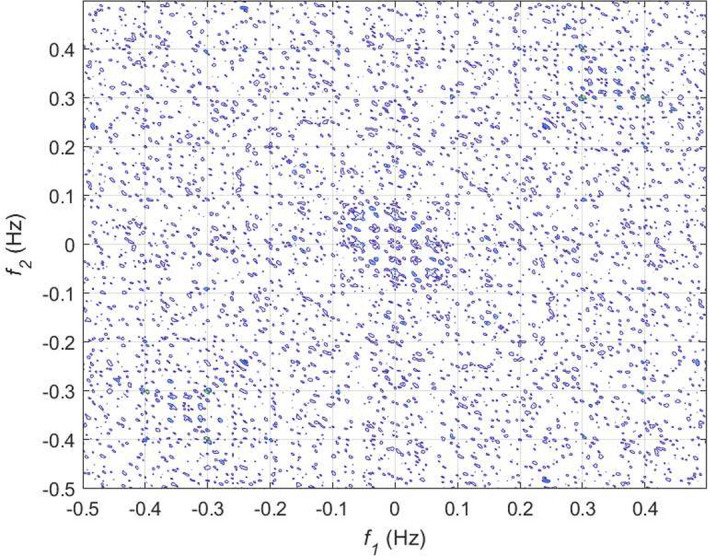
Bicoherence plot for HRV signal during meditative state

From the bispectrum analysis of HRV signals for meditative and pre-meditative states, Goshvarpour and Goshvarpour [34] have observed distinct differences in bispectrum amplitudes for the two states. They have observed an increase in mean bispectrum amplitude during meditation in case of KYM practitioners, while a significant decrease ($p<0.5$) in bispectrum amplitude during Chi meditation. However, their results are found to be containing bispectral components up to the range of $\sim$ 15 Hz, which is found to be exceeding the spectral bandwidth of HRV signal. This may also happen due to the consideration of higher sampling frequency for the signals in the study. From, bispectral analysis of the HRV signals as shown in Figs. 16 and 17, it can be observed that oscillations are more periodic during meditation. However, the power concentrations in the spectral bands are more evident from bicoherence plots as portrayed in Figs. 18 and 19. It can be seen that before meditation, prominent spectral components are present in both 0.05$-$0.15 Hz and 0.15$-$0.45 Hz spectral ranges. However, during meditation, prominent spectral components are found only in 0.05$-$0.1 Hz corresponding to LF band. It is to be mentioned that these two HOS-based plots are obtained by direct FFT technique. Multiple samples of length 256 are taken with an overlap of 50 samples, FFT length is chosen as 512 to estimate the bispectrum and bicoherence. In [84], Garcia et al. have corroborated that reduced HH power in a bispectrum analysis can be an indicator of alterations in vagal activity, which may drastically affect the psychological health by acting on the central autonomic network in response to psychological stress.

*Application of wavelet-based nonlinear analysis* The non-stationarity in HRV signal could easily lead to misleading information if segmentation-based analysis is not conducted. Traditional time domain and spectral analysis methods consider the whole signal to be a stationary one. Besides, many nonlinear methods are suitable to stationary signals only. Under such circumstances, wavelet analysis is well-designed to analyze such signals. It has the leverage of using a narrow wavelet for analyzing signals demanding high time-resolution and wider wavelet for those requiring high-frequency resolution [85]. A wavelet basically decomposes a signal into scale-specific waveforms by performing convolution operation with the dilated and translated wavelet function, $\psi (t)\in L^2({\mathbb {R}})$ known as mother wavelet. If *a* is the scaling parameter and *b* is the translation parameter, the wavelet transform of a signal *x*(*t*) is given by:

32 $$ W_{x}(a,b) = \frac{1}{\sqrt{a}}\int \limits _{-\infty }^{\infty }x(t)\psi ^*\left(\frac{t-b}{a}\right)dt. $$

Here, $*$ is the complex conjugate operator. The scaling parameter can be varied to change the width of the analyzing wavelet function and the shift parameter, *b* facilitates the location specific analysis.

On the other hand, the decomposition of a signal *x*[*n*] using discrete wavelet transform (DWT) for a wavelet function ($\psi [n]$), is given by:

33 $$\begin{aligned} W_{\phi }[j_0,k]=\frac{1}{\sqrt{N}}\sum _{n=1 }^{N}x[n]\phi _{j,k}[n],\\ W_{\psi }[j,k]=\frac{1}{\sqrt{N}}\sum _{n=1 }^{N}x[n]\psi _{j,k}[n]&\ \text { for}\ j\ge j_0,\\ \end{aligned} $$

where $\phi _{j,k}[n]=2^{j/2}\phi [2^{j}n-k]$, $\psi _{j,k}[n]=2^{j/2}\psi [2^{j}n-k]$ are sampled versions of scaling and wavelet functions, respectively; $j = 0,1,2,....J$ and $k = 0,1,2,...,2^J-1$ with $j_0$= 0 being the minimum scale, *J* the highest scale and *N* normally selected as integer power of 2. Decomposition of signals into different sub-bands with unique scales leads to detection of singularity under different scales, which makes it a proficient tool for multifractal analysis. This prompted researchers to analyze multifractality existing in various physical phenomena and contribute remarkable works [86, 87]. Besides, DWT and wavelet packet transform can yield various nonlinear metrics from their coefficients [88, 89].

Based on average wavelet coefficients (AWC), Papasimaki and Pallikari [32] have found that prominent peaks are obtained in the scales ranging from 8–32 beats during meditation. Their results indicates the presence of periodic behavior in LF region of HRV during meditation. Further, they observe a significant drop in Hurst exponent during meditation, which indicates destruction of long-range correlation behavior. In a study by Goshvarpour and Goshvarpour [90], daubechies wavelet ‘db4’ is applied to extract multiple features including the nonlinear parameters such as, skewness, kurtosis and entropy. They have achieved an accuracy of 99.61% in classifying the HRV of meditators (Chi and KYM) and non-meditators using probabilistic neural network (PNN).

## Discussion

We intend to present applications of various nonlinear dynamical approaches for studying HRV dynamics during meditation as reported in the literature over the last 20 years. It has been observed that due to the variation in meditation/relaxation and yoga techniques in terms of breathing procedures, duration and intensity, etc., their impact on ANS is different. To mention, slow breathing meditation and paced breathing (Kapalbhati) meditation adopt completely opposite procedure. Thanks to the PhysioNet that has provided standard datasets for studying the effects of meditation on a single platform [11–13, 15, 24, 27, 28, 30, 35, 37, 49]. However, we believe that availability of respiratory and blood pressure data could further enhance the depth of research in studying the impact of meditation. The rationale is that RSA also contributes spectral components at particular time scales which cause HRV modulation. Identically blood pressure also has impacts on HRV. It is to be noted that in case of classification-based works, exclusivity is a concern as majority of the works are based on binary classifications. Multiclass screening of different groups of subjects (viz., subjects under stress, exercise, meditator, runner, healthy subjects under resting condition) is probably much desired in present scenario.

From the literature it can be inferred that researchers have amply studied the variations in long-range correlation behavior of HRV, dynamical complexity in terms of regularity, and predictability/stability of the HRV signal. It is also noticed that there are some degree of differences in the research outcomes of some works possibly due to the use different datasets or some other reasons. It is observed that during meditation, a majority of researchers demonstrate significant decrease in the complexity of HRV signal by computing nonlinear parameters (entropy, CD, MED, LLE, Poincaré indices, etc.) [12, 13, 24, 26, 27, 29, 33], while a few have observed an increase in dynamical complexity [15, 28, 39]. To examine the regularity and complexity of HRV signals, various forms of entropy parameters are proposed. There are profound interest among researchers to use entropy markers in evaluating HRV signals representing well-being and different ailments; a few have worked on to find the most suitable entropy measure for small data size [26, 27, 38]. However, the notion of multiscale analysis is not yet explored pervasively in the studies related to meditation except in [10, 14]. The multiscale-based entropy analysis has revealed that meditative state is rather a complex state unlike that revealed by the entropy analysis at scale 1 [13, 26, 27, 38]. Besides entropy, fractal/multi-fractal analysis is also performed by many researchers [10, 15, 28, 29, 91]. In these studies, results are found to be inconsistent from one another. On applying VG, multifractality, and FDI techniques to HRV signals for meditative states, a significant increase in the complexity is observed [11, 28, 92]. On a nutshell, it can be stated that nonlinear study demonstrates that HRV possesses more periodic and decimated long-range correlation behavior, low fractal dimension, but higher dynamical complexity during meditation as compared to the non-meditative state. On the other hand, a prominent decrease in entropy, multifractality and dimensionality is observed in cardiac pathological conditions.

Moving on to other techniques, a new approach based on HRV auditory display is used in studying the impacts of meditation. In this technique, the HRV features (linear and nonlinear) are transformed to auditory signal pitch, timbres, etc., by a process known as sonification. On implementation of machine learning-based discrimination of meditative and pre-meditative state it has been noticed that only a few works [14, 27, 93, 94] employ machine learning techniques (pattern recognition, SVM, pNN, LVQ, etc., classifiers) to distinguish the meditative state from non-meditative state, probably due to the small size of publicly available datasets. However data augmentation strategy [14] such as window slicing, concatenation based techniques can be very effective to enable machine learning-based classification works; whereas, in studies related to cardiac pathologies, comparatively larger (adequate) amount data are available in public domain.

## Conclusion and future research trends

In this paper, we have carried out a brief review of works demonstrating scientific evidences of the impact of meditation and yoga on HRV dynamics under nonlinear domain. Nonlinear methods are chosen, since HRV is the output of nonlinear interactions of several sub-systems. Recent studies revealed that meditation is a complex state in which larger entropy values are found at higher scales. This corroborates the previous findings of higher LLE during meditation. One of the major limitations of the research on meditation and yoga is the non-availability of sufficient public labeled datasets for experimentation. Besides, given the fact that HRV signal often possess distinct temporal patterns over multiple scales, multiscale entropy/fractal analyses may divulge the hidden heartbeat dynamics in a more robust way. Some of the possible directions for future research in this topic could be:

1. No long-term analysis on the effects of different stages of meditation has been performed yet, using HRV signal before, during and after meditation, which may could help in tracking the transformation in ANS functionality over years of continuous practice.
2. Evaluation of variations in the dynamical complexity of HRV among experienced meditators, beginners, and control groups by taking sufficient experimental data. A study in this direction could provide more distinct and reliable outcomes.
3. To examine whether the changes in heart beat dynamics during meditation is due to controlled respiratory effort (RSA) or due to its notable effect on the ANS. This could be ascertained by using both the HRV and respiratory rate time series for analysis.

## Methods

This study is based on the information provided by scientific reports, articles indexed by PubMed, Google Scholar, Web of Science, and Scopus with different keywords, like, “Nonlinear HRV meditation”, “HRV nonlinear analysis yoga”, “HRV and ANS”, “autonomic regulation and nonlinear HRV”.

**Fig. 20:**
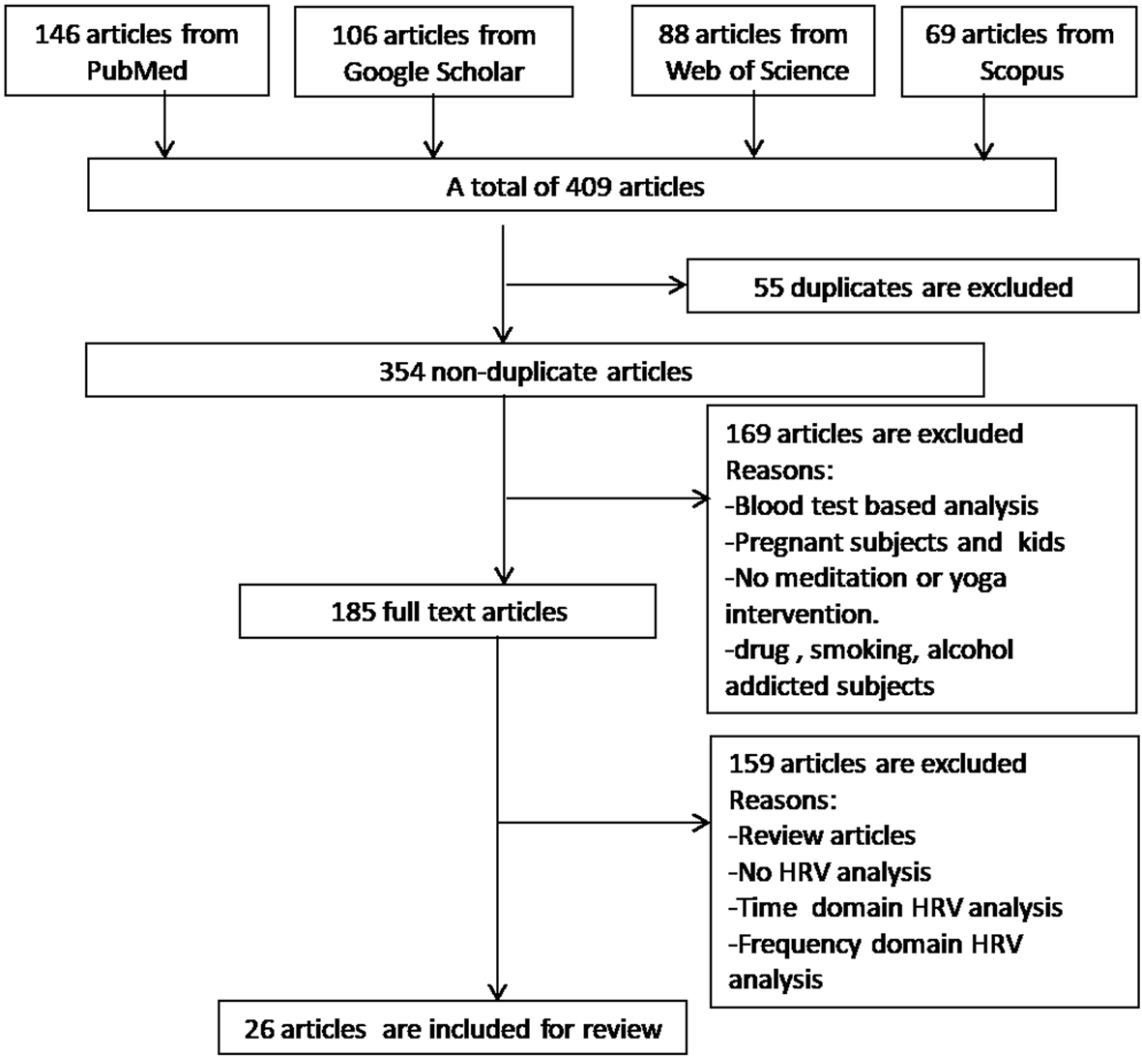
Criteria adopted for article selection

After preliminary investigation based on the inclusion–exclusion criteria detailed in Fig. 20, we have short-listed 26 research articles on nonlinear dynamical analysis of the HRV signal. Following this, we present analysis of the nonlinear methods that have gained much attention from the research community, while studying the HRV signal dynamics during meditation. Along with the theoretical background of various nonlinear methods of HRV analysis, we provide our perspectives supported by numerical simulations.

## Data Availability

Not applicable.

## Abbreviations

HRV: Hear rate variability
ANS: Autonomic nervous system
CHF: Congestive heart failure
CAD: Coronary artery disease
PNS: Parasympathetic nervous system
SNS: Sympathetic nervous system
SA: Sinoatrial
SDNN: Standard deviation of NN intervals
SDANN: SD of average NN intervals
RMSSD: Root mean square of successive differences between NN interval
HTI: HRV triangular index
TINN: Triangular interpolation of NN interval histogram
ANOVA: Analysis of variance
ANCOVA: Analysis of covariance
ULF: Ultra low frequency
VLF: Very low frequency
LF: Low frequency
HF: High frequency
FFT: Fast Fourier transform
PSD: Power spectral density
LLE: Lyapunov exponent
CD: Correlation dimension
NLS: Nonlinearity score
ShEn: Shannon entropy
CVD: Cardiovascular disease
GI: Guzik’s index
LOI: Line of identity
MSE: Multiscale entropy
HOS: Higher order spectra
ApEn: Approximate entropy,
CCTM: Component central tendency measures
DEA: Diffused entropy analysis
DFA: Detrended fluctuation analysis
DSJE: Double symbolic joint entropy
k: Degree of VG
LogEn: Log energy entropy
LZ: Lempel–Ziv
MED: Minimum embedding dimension
P(k): Degree distribution
PSVG: Power of scale-freeness in VG
SampEn: Sample entropy
